# Pathogenicity assessment of *Arcobacter butzleri* isolated from Canadian agricultural surface water

**DOI:** 10.1186/s12866-023-03119-x

**Published:** 2024-01-08

**Authors:** Izhar U. H. Khan, Wen Chen, Michel Cloutier, David R. Lapen, Emilia Craiovan, Graham Wilkes

**Affiliations:** 1 Ottawa Research and Development Centre (ORDC), Agriculture and Agri-Food Canada, 960 Carling Ave, Ottawa, ON K1A 0C6 Canada; 2 Natural Resources Canada, Ottawa, ON Canada

**Keywords:** *Arcobacter butzleri*, Antimicrobial resistance, Virulence-associated genes, ERIC-PCR, Canadian agriculture watershed, Surface water

## Abstract

**Background:**

Water is considered a source for the transmission of *Arcobacter* species to both humans and animals. This study was conducted to assess the prevalence, distribution, and pathogenicity of *A. butzleri* strains, which can potentially pose health risks to humans and animals. Cultures were isolated from surface waters of a mixed-use but predominately agricultural watershed in eastern Ontario, Canada. The detection of antimicrobial resistance (AMR) and virulence-associated genes (VAGs), as well as enterobacterial repetitive intergenic consensus-polymerase chain reaction (ERIC-PCR) assays were performed on 913 *A. butzleri* strains isolated from 11 agricultural sampling sites.

**Results:**

All strains were resistant to one or more antimicrobial agents, with a high rate of resistance to clindamycin (99%) and chloramphenicol (77%), followed by azithromycin (48%) and nalidixic acid (49%). However, isolates showed a significantly (*p* < 0.05) high rate of susceptibility to tetracycline (1%), gentamycin (2%), ciprofloxacin (4%), and erythromycin (5%). Of the eight VAGs tested, *cia*B, *mvi*N, *tly*A, and *pld*A were detected at high frequency (> 85%) compared to *irg*A (25%), *hec*B (19%), *hec*A (15%), and *cj*1349 (12%) genes. Co-occurrence analysis showed *A. butzleri* strains resistant to clindamycin, chloramphenicol, nalidixic acid, and azithromycin were positive for *cia*B, *tly*A, *mvi*N and *pld*A VAGs. ERIC-PCR fingerprint analysis revealed high genetic similarity among strains isolated from three sites, and the genotypes were significantly associated with AMR and VAGs results, which highlight their potential environmental ubiquity and potential as pathogenic.

**Conclusions:**

The study results show that agricultural activities likely contribute to the contamination of *A. butzleri* in surface water. The findings underscore the importance of farm management practices in controlling the potential spread of *A. butzleri* and its associated health risks to humans and animals through contaminated water.

## Highlights

1. *Arcobacter butzleri* was highly prevalent in agricultural surface water.

2. Strains were highly resistant to antimicrobial agents.

3. Virulence genes were detected at a high frequency.

4. High genetic similarity and association among strains from various sites.

## Background

Over the past few decades, it has been determined that untreated water is an important source of *Arcobacter* species which are considered emerging human pathogens causing enteritis [1, 2]. *Arcobacter* species are gram-negative and are considered the fourth most important pathogenic member of the *Campylobacteraceae* family [3, 4]. Among these, *A. butzleri*, *A. cryaerophilus*, and *A. skirrowii* have been isolated from humans and animals [1]. *A. butzleri* is known to cause various human infections making it clinically important. *A. butzleri* strains isolated from human and animal sources have been reported and classified as emerging human pathogens (EHPs) by the International Commission on Microbiological Specifications for Foods [5].

*A. butzleri* and *A. cryaerophilus* can cause abdominal pain, gastroenteritis and acute diarrhea or prolonged watery diarrhea in humans. They can even cause bacteremia, abortion and mastitis in cattle and swine [6–8]. The main sources of *A. butzleri* transmission include ingestion of contaminated water, food contamination, and fecal shedding from livestock such as cattle [1, 7]. Uncooked or minimally processed foods also pose a high risk in the transmission of infection and can consequently be a serious hazard to public health [9]. Since contaminated water is considered one of the possible routes of infection in humans and animals [7], examining the prevalence of *Arcobacter* species in water is useful to better understand the source and transmission process of these pathogens [10].

Many *Arcobacter* species are known to acquire resistance against a wide range of commonly used antibiotics due to lateral gene transfer; hence, various antimicrobial agents are proven ineffective in managing *A. butzleri* infections [7, 11]. The antimicrobial resistance (AMR) profile of *A. butzleri* previously reported has shown high resistance against erythromycin and ciprofloxacin, which are commonly used antibiotics for treating *Campylobacter* infections. However, some studies have revealed AMR in *A. butzleri* against amoxicillin, nalidixic acid, gentamicin, clindamycin, azithromycin, ciprofloxacin, metronidazole, carbenicillin, and cefoperazone, but maximum susceptibility to fluoroquinolones and tetracycline; hence, these two antibiotics can be used to treat infections caused by *A. butzleri* [12–15]. Notably, certain *Arcobacter* isolates have exhibited resistance to multiple drugs (known as multidrug resistance, MDR), including trimethoprim and cephalosporins, as previously reported [7, 16, 17]. Further investigations have revealed that an increase in the degree of antimicrobial resistance in *A. butzleri* strains may be attributed to their exposure to antibiotics commonly used in veterinary and public health applications as documented by Luangtongkum et al. [18].

To fully comprehend the pathogenicity of *A. butzleri*, it is important to investigate its virulence factors, such as adhesion, invasion, and cytotoxic capacity. However, current knowledge on these mechanisms and potential virulence factors remains insufficient [19–21]. Pathogens with certain virulence genes could potentially lead to infections in humans and other animals [22, 23]. Analysis of the *A. butzleri* RM 4018 genome has revealed the presence of several putative virulence genes, including *cad*F, *cj*1349, *cia*B, *mvi*N, *pld*A, *tly*A, *irg*A, *hec*A, and *hec*B. The gene *mvi*N encodes protein MViN, which is essential for peptidoglycan biosynthesis [24]. Similarly, in *Campylobacter* species, *cad*F and *cj*1349 encode fibronectin-binding proteins that enhance bacterial attachment to the host cells [25, 26]. *Campylobacter* invasive antigen B (*cia*B) is involved in host cell invasion [27]. The gene *hec*A, a member of the filamentous hemagglutinin family, encodes protein HecA (Rojas), while *hec*B encodes haemolysin activation protein [28], and *tly*A encodes hemolysin. Notably, hemolysin proteins are also present in other human infectious agents, such as *Mycobacterium tuberculosis* and *Serpulina* [29]. The *irg*A gene encodes an iron-regulated outer membrane protein (IrgA) that confers pathogenicity to *E. coli* [30, 31], while *pld*A encodes an outer membrane phospholipase PldA that mediates hemolysis of erythrocytes in humans [32]. However, it remains unclear whether these putative virulence factors function similarly in other bacterial pathogens.

DNA fingerprinting techniques, such as random amplification of polymorphic DNA (RAPD), restriction fragment length polymorphism (RFLP), pulsed-field gel electrophoresis (PFGE) and enterobacterial repetitive intergenic consensus-polymerase chain reaction (ERIC-PCR), are commonly used to study genotypic diversity of bacterial pathogens [33–35]. Of these methods, ERIC-PCR has a higher discriminatory power and can produce more consistent and intricate results than the other techniques [36, 37]. Moreover, ERIC-PCR is more effective and efficient method for strain-level genotyping. ERIC sequences, alternatively known as intergenic repetitive units, have been used in several studies to investigate the level of genetic variation and relatedness among pathogenic bacterial strains or species [36]. The intergenic repetitive units specifically correlated with the level of heterogeneity observed in *Arcobacter* species was likely the outcome of genetic recombination between progeny of parent genotypes [37]. Bacterial pathogens are known to be continuously evolving, and thus a high level of genetic diversity is expected in the case of *A. butzleri*.

Therefore, this study was initiated with the aim of investigating the degree of genetic diversity, prevalence of virulence genes, and status of antimicrobial resistance in *A. butzleri* strains isolated from surface water samples collected from different sites in a mixed-use but predominately agricultural watershed in eastern Ontario, Canada.

## Results

### Detection and identification of *Arcobacter *species and *A. butzleri* by multiplex PCR analysis

Of the total 797 surface water samples, 2040 strains isolated from 11 sites with agricultural-dominated upstream land uses were confirmed as *Arcobacter* using genus-specific PCR assay. The sites with *Arcobacter* species isolated include Site 1 (*n* = 205; 10%), 5 (*n* = 645; 32%), 6 (*n* = 357; 18%), 9 (*n* = 514; 25%), 15 (*n* = 264; 13%), 17 (*n* = 1; 0.04%), 18 (*n* = 6; 0.3%), 19 (*n* = 12; 0.6%), 20 (*n* = 28; 1.4%), 22 (*n* = 2; 0.1%), and 23 (*n *= 6; 0.3%). Of these 2040 strains, 913 (48%) isolates from Sites 1 (*n* = 98; 11%), 5 (*n* = 279; 31%), 6 (*n* = 154; 17%), 9 (*n* = 247; 27%), 15 (*n* = 102; 11%), 17 (*n* = 1; 0.1%), 18 (*n* = 2; 0.2%), 19 (*n* = 5; 0.5%), 20 (*n* = 17; 2%), 22 (*n* = 2; 0.2%), and 23 (*n* = 6; 0.6%) were identified as *A. butzleri* by species-specific multiplex PCR assay. These *A. butzleri* isolates were subjected to characterization of Antimicrobial Resistance (AMR), Virulence-associated Genes (VAGs) detection, and genotyping using ERIC-PCR assays.

### Antimicrobial resistance analysis

Of the total 913 *A butzleri* isolates, the majority were resistant to clindamycin (99%), followed by chloramphenicol (77%), nalidixic acid (49%), and azithromycin (48%) (Table 1). However, small proportion of the isolates displayed resistance to erythromycin (5%), ciprofloxacin (4%), gentamycin (2%) and tetracycline (1%). However, a significantly (*p* < 0.05) high level of resistance to clindamycin was observed in *A. butzleri* strains isolated from sites 6 (98%), 5 (97%), 15 (93%), 9 (90%) and 1 (88%). Similarly, sites 6 (53%), 5 (51%), and 9 (49%) showed high resistance to azithromycin. The frequency of isolates resistant to chloramphenicol was highest at sites 5 (86%) and 6 (82%) and relatively lower at sites 9 (73%), 15 (73%), and 1 (57%). Resistance against nalidixic acid was found most prevalent in isolates from sites 6 (58%) and 9 (54%), followed by sites 5 (47%) and 15 (45%). Overall, the detection frequency of antimicrobial-resistant isolates was significantly (*p* < 0.05) high at site 6, where a high level of resistance to all antimicrobial agents was observed. By contrast, many isolates from site 1 showed susceptibility to erythromycin, tetracycline, ciprofloxacin, and gentamycin. Sites 17 to 23, with a very limited number of isolates, showed 100% resistance to clindamycin, chloramphenicol, azithromycin and nalidixic acid. However, all isolates from sites 22 and 23 showed resistance to clindamycin and chloramphenicol as compared to isolates from sites 19 and 20 which showed (100%) resistance to clindamycin.

**Table 1:** Number (percentage) of *A. butzleri* isolates resistant to antimicrobial agents

Sampling sites	Total number of isolates	Number of antimicrobial resistant strains (%)							
		**Azi**^a^	**Chl**	**Cli**	**Nal**	**Cip**	**Ery**	**Gen**	**Tet**
1	98	41 (42)	56 (57)	86 (88)	33 (34)	7 (7)	7 (7)	7 (7)	6 (6)
5	279	141 (51)	240 (86)	271 (97)	131 (47)	5 (2)	11 (4)	5 (2)	0 (0)
6	154	82 (53)	126 (82)	151 (98)	90 (58)	6 (4)	10 (6)	6 (4)	0 (0)
9	247	122 (49)	180 (73)	282 (90)	134 (54)	11 (4)	13 (5)	0 (0)	0 (0)
15	102	46 (45)	74 (73)	95 (93)	46 (45)	4 (4)	7 (7)	0 (0)	0 (0)
17	1	1 (100)	1 (100)	1 (100)	1 (100)	0 (0)	0 (0)	0 (0)	0 (0)
18	2	0 (0)	2 (100)	2 (100)	2 (100)	0 (0)	0 (0)	0 (0)	0 (0)
19	5	2 (20)	3 (60)	5 (100)	2 (20)	0 (0)	0 (0)	0 (0)	0 (0)
20	17	3 (18)	10 (59)	17 (100)	7 (41)	0 (0)	0 (0)	0 (0)	0 (0)
22	2	0 (0)	2 (100)	2 (100)	0 (0)	0 (0)	0 (0)	0 (0)	0 (0)
23	6	1 (17)	6 (100)	6 (100)	0 (0)	0 (0)	0 (0)	0 (0)	0 (0)
Total	913	439 (48)	700 (77)	901 (99)	446 (49)	33 (4)	48 (5)	18 (2)	6 (1)

^a^*Azi* Azithromycin, *Chl* Chloramphenicol, *Cli* Clindamycin, *Nal* Nalidixic acid, *Cip* Ciprofloxacin, *Ery* Erythromycin, *Gen* Gentamycin, *Tet* Tetracycline

Moreover, *A. butzleri* isolates (*n* = 786; 86%) showed significantly (*p* < 0.05) high multi-drug resistance (MDR), particularly in isolates from sites 5 (93%), 6 (88%), and 15 (86%) as compared to sites 9 (82%) and 1 (72%). Whereas 61% (*n* = 553) strains showed resistance to four or more antibiotics, while 33% (*n* = 303) isolates showed resistance to nalidixic acid and one of the following antimicrobials, azithromycin, chloramphenicol, or clindamycin. However, 1% of the isolates from sites 6 and 9 showed resistance to all eight antimicrobials tested. In addition, 4% (*n* = 37) of the total isolates from all sites were susceptible to all eight antimicrobials tested.

### Detection and distribution of VAGs

Overall, all eight VAGs were detected in 913 *A. butzleri* strains at a variable frequency (Table 2), with *cia*B (89%) and *tly*A (88%) being most prevalent, followed by *pld*A (87%) and *mvi*N (86%) genes. However, *cj*1349 (12%), *hec*A (15%), *hec*B (19%) and *irg*A (25%) genes were detected at relatively low frequency. Among eight VAGS, *cia*B, *mvi*N, *tly*A and *pld*A genes were detected significantly (*p* < 0.05) high across all 11 sites. Whereas no significant (*p* > 0.05) difference, in the rate of prevalence of *cj*1349, *hec*B, *hec*A and *irg*A VAGs, across sites was observed. Among these eight VAGs, *tly*A (94%) and *mvi*N (90%) were detected in isolates from site 1 as compared to the *cia*B (94%) and *pld*A (89%) genes detected in isolates from site 15. Overall, isolates from site 15 had the highest (> 88%) prevalence of *cia*B, *mvi*N, *tly*A and *pld*A genes. On the other hand, a high prevalence of *hec*B and *irg*A genes were detected in site 6, as compared to sites 1 and 9 isolates that showed a high prevalence of *cj*1349 and *hec*A genes. Although sites 17 to 23 had limited isolates, *cia*B, *tly*A, *mvi*N, *hec*B, *pld*A and *irg*A genes were detected in site 17 isolates as compared to sites 18 and 19 where all isolates were also positive for *cia*B, *tly*A, *mvi*N and *pld*A genes. Whereas, all isolates from site 20 were positive for *tly*A and *mvi*N as compared to site 22, positive for *tly*A, *mvi*N and *pld*A genes. Interestingly, only *cia*B gene was detected in all isolates from site 23 (Table 2).

**Table 2:** Number (percent) VAGs detected in *A. butzleri* isolates

Sampling sites	Total number of isolates	Number of VAGs positive isolates (%)							
		***cia*B** **(284 bp)**	***cj*1349** ***(659 bp)***	***tly*A** **(230 bp)**	***mvi*N** **(294 bp)**	***hec*B** **(528 bp)**	***pld*A** **(293 bp)**	***irg*A** **(437 bp)**	***hec*A** **(537 bp)**
1	98	82 (84)	18 (18)	92 (94)	88 (90)	11 (11)	86 (88)	15 (15)	5 (5)
5	279	248 (89)	34 (12)	242 (87)	238 (85)	54 (19)	245 (88)	86 (31)	51(18)
6	154	139 (90)	22 (14)	137 (90)	135 (88)	57 (37)	136 (88)	73 (47)	21 (14)
9	247	227 (92)	25 (10)	222 (90)	203 (89)	31 (13)	214 (87)	29 (12)	47 (19)
15	102	96 (94)	11 (11)	94 (92)	94 (92)	19 (19)	91 (89)	23 (23)	11(11)
17	1	1 (100)	0 (0)	1 (100)	1 (100)	1 (100)	1 (100)	1 (100)	0 (0)
18	2	2 (100)	0 (0)	2 (100)	2 (100)	0 (0)	2 (100)	0 (0)	0 (0)
19	5	5 (100)	1 (20)	5 (100)	5 (100)	0 (0)	5 (100)	4 (80)	1 (20)
20	17	9 (53)	0 (0)	17 (100)	17 (100)	3 (18)	8 (47)	0 (0)	0 (0)
22	2	0 (0)	0 (0)	2 (100)	2 (100)	0 (0)	2 (100)	0 (0)	0 (0)
23	6	6 (100)	0 (0)	4 (67)	2 (33)	0 (0)	1 (17)	1 (17)	0 (0)
Total	913	815 (89)	111 (12)	801 (88)	785 (86)	176 (19)	791 (87)	232 (25)	136 (15)

For analyzing the occurrence of multi-VAGs, 889 (97%) *A. butzleri* strains were detected positive with a significantly high prevalence (*p* < 0.05) in sites 1 and 6 (99%) than sites 9 and 15 (98%) and 5 (96%), respectively. The majority of isolates (39%) had four or more VAGs with the combination of *cia*B, *tly*A, *mvi*N and *pld*A genes. Interestingly, all eight VAGs were harbored only in two of the isolates from sites 9 and 5. Overall, 14 of the isolates, mostly from sites 1 and 5, were detected without any VAGs.

### Co-occurrence of AMR and VAGs in *A. butzleri* strains

Overall, at a significance level of 0.05, *A. butzleri* strains from different sites that had resistance to clindamycin, chloramphenicol, nalidixic acid, and azithromycin were also detected positive for *cia*B, *tly*A, *mvi*N and *pld*A genes. Whereas isolates that showed resistance to clindamycin were also positive for *tly*A and *mvi*N genes. *A. butzleri* isolates from sites 6 and 15 that showed resistance towards nalidixic acid were also resistant to azithromycin also detected positive for *cia*B and *thy*A genes. The strains resistant to clindamycin and chloramphenicol were also positively correlated to the *tly*A and *mvi*N genes. Interestingly, *mvi*N and *hec*A did not show co-occurrence patterns with each other in the tested isolates. On the other hand, *mvi*N showed a co-occurrence pattern with *pld*A; however, *cj*1349 gene did not show a co-occurrence pattern with them. Isolates from sites 5 and 9 positive for *cia*B and *tly*A were frequently detected with *mvi*N and *pld*A genes. Despite the site of isolation of *A. butzleri*, the isolates showed a high frequency of resistance to the clindamycin, chloramphenicol, nalidixic acid, and azithromycin were also positive for *cia*B, *tly*A, *mvi*N and *pld*A genes, respectively.

### ERIC-PCR profiling of *A. butzleri* strains

**Fig. 1:**
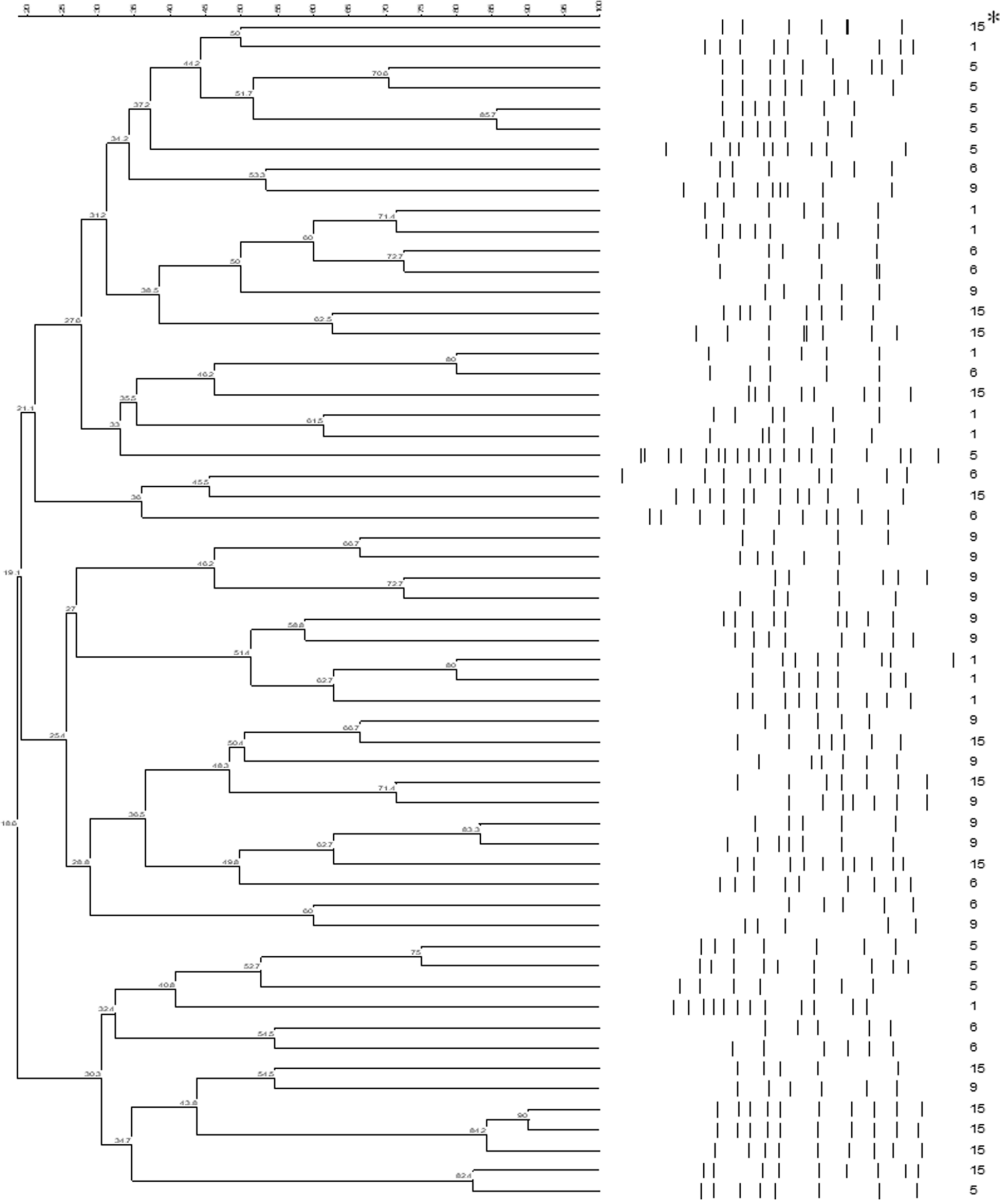
Dendrogram analysis showing ERIC-PCR based distinct band patterns of *A. butzleri* strains isolated from surface water samples collected from an agricultural watershed. * denotes various agricultural sampling sites

The ERIC-PCR fingerprinting pattern of *A*. *butzleri* strains from different sites were examined using cluster analysis. Based on theERIC-PCR band patterns (Fig. 1), with a discriminatory index 0.894, a total of 913 *A. butzleri* genotypes were distinguished. These isolates were grouped into 38 clusters using a 30% similarity cut-off. Overall, a total of 328 (36%) genotypes that were isolated from sites 1, 5, 6, 9 and 15 showed high similarity. Of these isolates, 201 derived from sites 5 and 9 were grouped with strains from site 15. Isolates from site 1 were grouped into 3 clusters, represented by 10 to 39 isolates, of which, 27 genotypes showed > 40% homogeneity. Isolates from site 5 formed 6 clusters; each was represented by 21 to 100 isolates. Of the six clusters, 68 genotypes had > 40% homogeneity. Isolates from site 6 were grouped into eight main clusters and each was represented by 5 to 35 isolates, with 45 genotypes showing > 20% homogeneity. At site 9, a total of eight clusters were observed and each was represented by 6 to 39 isolates, where 52 genotypes showed > 35% homogeneity. Moreover, isolates from site 15 formed five clusters and each was represented by 7 to 35 isolates. At site 15, 16 genotypes showed > 30% homogeneity. Interestingly, the highest similarity of genotypes was detected in isolates from sampling sites 5, 9 and 15, which indicates that the water contamination may occur from the same sources.

### Co-occurrence of ERIC-PCR and AMR

Strains that showed similar ERIC-PCR profiles from site 1 exhibited remarkable resistance to clindamycin (94%) and chloramphenicol (85%), in contrast to lower resistance levels to azithromycin (73%) and nalidixic acid (42%), respectively. Similarly, isolates from site 5 were highly resistant to clindamycin (92%) and chloramphenicol (77%), with reduced resistance to azithromycin (66%) and nalidixic acid (50%). Strains from site 6 displayed the highest resistance to chloramphenicol (89%) and clindamycin (84%), as opposed to nalidixic acid (51%) and azithromycin (49%). Moreover, site 9 isolates showed high resistance to clindamycin (94%) and chloramphenicol (88%), compared to nalidixic acid (66%) and azithromycin (49%). Lastly, site 15 isolates showed the highest resistance to each of clindamycin and chloramphenicol (68%), followed by azithromycin (30%) and nalidixic acid (20%) (Table 3).

**Table 3:** Number (percentage) of isolates showing similar ERIC-PCR and AMR patterns

Site	Number of isolates similar ERIC-PCR pattern	Number of antimicrobial-resistant isolates (%)							
		**Azi**	**Chl**	**Cli**	**Nal**	**Cip**	**Ery**	**Gen**	**Tet**
1	52^a^	38 (73)	44 (85)	49 (94)	22 (42)	4 (8)	2 (4)	0 (0)	2 (4)
5	132	87 (66)	102 (77)	121 (92)	66 (50)	6 (5)	6 (5)	3 (2)	1 (1)
6	80	32 (40)	71 (89)	67 (84)	41 (51)	3 (4)	5 (6)	1 (1)	0 (0)
9	140	69 (49)	123 (88)	131 (94)	92 (66)	3 (2)	7 (5)	2 (1)	3 (2)
15	76	23 (30)	52 (68)	52 (68)	15 (20)	1 (1)	1 (1)	0 (0)	2 (3)
17	0	0 (0)	0 (0)	0 (0)	0 (0)	0 (0)	0 (0)	0 (0)	0 (0)
18	0	0 (0)	0 (0)	0 (0)	0 (0)	0 (0)	0 (0)	0 (0)	0 (0)
19	2	0 (0)	1 (50)	2 (100)	0 (0)	0 (0)	0 (0)	0 (0)	0 (0)
20	2	0 (0)	2 (100)	2 (100)	0 (0)	0 (0)	0 (0)	0 (0)	0 (0)
22	0	0 (0)	0 (0)	0 (0)	0 (0)	0 (0)	0 (0)	0 (0)	0 (0)
23	2	1 (50)	2 (100)	2 (100)	0 (0)	0 (0)	0 (0)	0 (0)	0 (0)

^a^Number of positive isolates

Conversly, isolates from all sites showed low to no resistance to ciprofloxacin, erythromycin, gentamycin and tetracycline. Isolates from sites 17, 18 and 20 exhibited dissimilar ERIC-PCR profiles, whereas sites 20 and 23 displayed 100% resistance to clindamycin and chloramphenicol. However, site 19 isolates showed only demonstrated 100% resistance to clindamycin alone. Isolates with ERIC-PCR patterns displaying greater than > 80% homogeneity in cluster analysis demonstrated the highest rates of MDR in isolates at sites 1 (79%) and 9 (70%), in contrast to site 5 (58%) and sites 6 and 15 (both at 55%). In contrast, strains from sites 17 to 23 showed no MDR. A notable presence of resistance to a combination of clindamycin, chloramphenicol, and azithromycin was observed in these isolates (Table 3).

### Co-occurrence of ERIC-PCR pattern and VAGs

The *cia*B gene was found most prevalent at sites 6 (96%) and 5 and 9 (both at 87%) compared to sites 1 (79%) and 15 (67%). Isolates from site 6 exhibited high prevalence of the *mvi*N gene (81%), followed by sites 9 (75%), 5 (74%), 15 (58%) and 1 (38%). Conversly, *tly*A was more prevalent at sites 9 (94%) and 6 (85%) compared to sites 5 (78%), 1 (73%) and 15 (64%). The *pld*A gene was most prevalent at site 6 (74%), followed by sites 9 (70%), 5 (54%), 15 (43%) and 1 (33%). The *cj*1349, *irg*A, *hec*B and *hec*A genes were present in fewer than 15% of isolates. Moreover, isolates with similar ERIC-PCR patterns showing greater than 80% homogeneity had a high prevalence of multi-VAGs (three or more) at sites 6 and 9 (59%), 1 (56%), followed by sites 15 (47%) and 5 (6%) isolates. However, no isolates with multi-VAGs were found at sites 17 to 23 (Table 4).

**Table 4:** Number (percentage) of isolates showing similar ERIC-PCR and VAGs patterns

Sampling sites	Similar ERIC-PCR pattern	Number of VAG positive isolates (%)							
		***cia*B**	***cj*1349**	***tly*A**	***mvi*N**	***hec*B**	***pld*A**	***irg*A**	***hec*A**
1	52^a^	41 (79)	3 (6)	38 (73)	20 (38)	5 (10)	17 (33)	2 (4)	3 (6)
5	132	115 (87)	11 (8)	103 (78)	98 (74)	11 (8)	71 (54)	7 (5)	9 (7)
6	80	77 (96)	6 (8)	68 (85)	65 (81)	4 (5)	59 (74)	1 (1)	0 (0)
9	140	122 (87)	21 (15)	131 (94)	105 (75)	11 (8)	98 (70)	5 (4)	7 (5)
15	76	51 (67)	2 (3)	49 (64)	44 (58)	3 (4)	33 (43)	0 (0)	2 (3)
17	0	0 (0)	0 (0)	0 (0)	0 (0)	0 (0)	0 (0)	0 (0)	0 (0)
18	0	0 (0)	0 (0)	0 (0)	0 (0)	0 (0)	0 (0)	0 (0)	0 (0)
19	2	2 (100)	0 (0)	2 (100)	0 (0)	0 (0)	1 (50)	0 (0)	0 (0)
20	2	2 (100)	0 (0)	2 (100)	0 (0)	0 (0)	0 (0)	0 (0)	0 (0)
22	0	0 (0)	0 (0)	0 (0)	0 (0)	0 (0)	0 (0)	0 (0)	0 (0)
23	2	2 (100)	0 (0)	2 (100)	1 (50)	0 (0)	0 (0)	0 (0)	0 (0)

^a^Number of positive isolates

Genotypically, of the 52 isolates from site 1 with similar ERIC-PCR patterns, 37 (71%) showed 100% similarity in cluster analysis, and of those, 5 (10%) isolates exhibited similar AMR and VAGs profiles. From site 5, 98 of 132 isolates (74%) showed 100% similarity, with 19 (14%) similar AMR and VAGs profiles. At site 6, 51 of 80 isolates (64%) had 100% similarity with 7 (9%) isolates demonstrated similar AMR and VAGs profiles. At site 9, 103 of 140 isolates (74%) showed 100% similarity with 21 (15%) showing similar AMR and VAGs profiles. For site 15 isolates, 63 (83%) of 76 displayed 100% homogeneity, and 11 (15%) had similar AMR and VAGs profiles. Sites 17,18 and 22, displayed dissimilar ERIC-PCR patterns, whereas sites 18, 20 and 23 had similar ERIC-PCR patterns but did not achieve 100% homogeneity in cluster analysis (Table 5).

**Table 5:** Number (percent) of *A. butzleri* isolates showing similar ERIC-PCR, AMR and VAGs patterns in cluster analysis

Site	Similar ERIC-PCR pattern	Cluster analysis (similarity 100%) (%)	Isolates with AMR and VAGs (%)	Multidrug-resistance (similarity > 80%) (%)	Multi-VAGs (similarity > 80%) (%)
1	52^a^	37 (71)	5 (10)	41 (79)	29 (56)
5	132	98 (74)	19 (14)	77 (58)	81 (6)
6	80	51 (64)	7 (9)	44 (55)	47 (59)
9	140	103 (74)	21 (15)	98 (70)	82 (59)
15	76	63 (83)	11 (15)	42 (55)	36 (47)
17	0	0 (0)	0 (0)	0 (0)	0 (0)
18	0	0 (0)	0 (0)	0 (0)	0 (0)
19	2	0 (0)	0 (0)	0 (0)	0 (0)
20	2	0 (0)	0 (0)	0 (0)	0 (0)
22	0	0 (0)	0 (0)	0 (0)	0 (0)
23	2	0 (0)	0 (0)	0 (0)	0 (0)

^a^Number of positive isolates

Interestingly, seven isolates from site 9 showed high similarity to four isolates from site 15, showed 100% similarity cluster analysis and had similar AMR and VAGs profiles. Moreover, five isolates from site 1 were found 100% similar to three isolates from site 6 in cluster analysis and exhibited similar AMR and VAGs profiles, where these isolates also exhibited MDR and multi-VAGs profiles.

## Discussion

A high number (∼63%) of human infections are due to the consumption of or close contact with *A. butzleri* contaminated water [1, 38]. In the present study, 45% of the total isolates from agricultural surface water were identified as *A. butzleri*. Of eleven stream sites, sites 1, 5, 6, 9 and 15 demonstrated the highest prevalence of *A. butzleri*. Particularly, site 15 is located in a smaller stream order and possibly impacted by drainages from septic systems of nearby houses and livestock in close proximity to water ways. The other sites, located in medium to large streams, might have additional contamination sources such as wildlife (migratory birds) and urban runoff. Although the rate of isolation of *Arcobacter* species varies significantly globally, 20.8% of water samples were found positive for *Arcobacter* species with 90% identified as *A. butzleri*, as reported by Laishram et al. [37]. Another study showed a relatively lower prevalence of *A. butzleri* in creeks and streams, with no detection in ponds and drinking water samples [10]. However, in the current study, a moderate (45%) frequency was observed. The isolation rate can be influenced by factors such as sampling and isolation methods, ecological and geographical characteristics of sampling sites, and water pollution mechanisms [39–41]. Many studies have suggested that the abundance of *A. butzleri* in water resources is due to their high viability in water and a potential competitive inhibitory effect on the other species [41]. The presence of *Arcobacter* species in water resources signals a possible risk of transmission to humans and animals, as well as the potential for contamination of food products.

The phenotypic characterization, including antimicrobial resistance of *A. butzleri* isolates, demonstrates that isolates from these 11 sites have developed resistance to clindamycin (99%), chloramphenicol (77.7%), azithromycin (48.8%) and nalidixic acid (47.4%). AMR patterns observed were comparable with a previous study where *A. butzleri* was isolated from different environmental aquatic sources [12]. However, the acquired resistance to clindamycin is particularly concerning because this antibiotic is the first-line drug for treating *Campylobacter* infection in humans [13, 15]. Similarly, azithromycin and ciprofloxacin are widely used for human infections [13]. Notably, all strains in our study were susceptible to tetracycline (1%), gentamycin (2%), ciprofloxacin (4%), and erythromycin (5%). Erythromycin and gentamicin are also frequently used as alternative treatments for campylobacteriosis [13, 15]. Thus, ciprofloxacin, tetracycline, erythromycin, and gentamycin are recommended as the first-choice antibacterial agents, with erythromycin as a close second for the treatment of *Arcobacter*-associated infections in humans and animals. A plausible explanation for the emergence and spread of clinical antibiotic resistance found in the isolates could be attributed to the unrestricted usage of these antibiotics in agriculture, as suggested by Chang et al [42]. This study supports the notion that ciprofloxacin, tetracycline, gentamycin, and erythromycin could be the antibiotics of choice for treating other pathogens in agriculture and animal husbandry practices where antimicrobial resistance is prevalent [42].

In our study, a high frequency (33%) of resistance to a combination of four tested antimicrobials (azithromycin, chloramphenicol, clindamycin, and nalidixic acid) was observed, with 1% of the *A. butzleri* population resistant to all eight antimicrobials tested. Previous studies have reported a variable frequency of MDR (ranging from 20 to 72%) among *A. butzleri* strains isolated from human and animal sources [15, 38, 43–45]. Wastewater from industrial plants, healthcare services, and agriculture are point sources for antimicrobials, antibiotic-resistant bacteria, and antibiotic resistance genes [46, 47]. The presence of *A. butzleri* in various water sites suggests the role of these isolates in the environment and food chain contamination, posing a potential threat to public health.

In various studies, *Arcobacter* VAGs have been detected in humans, animals, and food samples [17, 19–22, 37, 48, 49]. Information on the virulence properties of potentially pathogenic *A. butzleri* remains scarce, but some studies have elucidated the distribution of virulence markers and the adhesive, invasive and toxicity capacity of arcobacters, highlighting its high invasion ability to the host cells [21]. Although several genes have already been identified in the *A. butzleri* genome, it is still unclear if the putative virulence factors have similar functions in other microbial species [28]. The eight VAGs in *A. butzleri* isolates were detected at a variable frequency; four of these, *cia*B, *mvi*N, *tly*A and *pld*A were predominantly (> 86%) observed. Similar results were reported by Douidah et al. [19], which showed a 93% prevalence for the *cia*B and *mvi*N VAGs. However, low prevalence (< 25%) was noted for *cj*1349, *hec*A, *hec*B, and *irg*A VAGs across all tested isolates, consistent with previous studies [19, 20]. However, Rathlavath et al. [50] demonstrated a high (97%) prevalence of *cj*1349 gene in *A. butzleri* strains isolated from coastal water, fish and shellfish samples, which contradicts to our study results. This discrepancy could be due to the origin of the isolates. The high prevalence of *cia*B, *tly*A, *mvi*N and *pld*A VAGs in all isolates also demonstrates a correlation between the presence of these VAGs and infections in humans and animals. Overall, it can be concluded that the common distribution pattern of VAGs among *A. butzleri* isolates collected from various water sites may have the same pathogenic potential as known for strains isolated from various food and fecal sources. Detailed data analysis demonstrated the prevalence of multiple VAGs (ranging from 2 to 8) in most isolates (97%), with a considerable percent (54%) having four or more VAGs. These results are in accordance with an earlier study by Rathlavath et al. [50]. While research on the roles of these VAGs in *A. butzleri* continues, the data from this study could be useful in understanding their pathogenic potential and associated health risks to humans and animals.

Since the relationship between resistance and virulence in arcobacters is still unclear, it is often presumed that MDR arcobacters with more antimicrobial drug resistance account for greater virulence [51]. This could be a possible misconception, as it greatly relies on the phylogenetic lineage and resistant determinants of the specific isolate. This study is the first to demonstrate a correlation between AMR and VAGs. Therefore, the association and co-occurrence of AMR and VAGs in *A. butzleri* strains isolated from various agricultural sites were critically analyzed. Exploring this correlation in other studies could be helpful in managing diseases associated with *A. butzleri*. The co-occurrence analysis revealed a positive relationship between resistance to azithromycin, chloramphenicol, clindamycin, and nalidixic acid and the presence of *cia*B, *mvi*N, *tly*A and *pld*A VAGs. In a previous study, a similar positive correlation between the presence of AMR and VAGs in *E. coli* and *E. faecalis* as well as *S. aureus* and *S. pneumoniae* were reported [52–54]. However, a negative correlation was observed for the presence of tetracycline and *hec*A VAG in our isolates, in sites 17, 18, 19, 20, 22, and 23 that had fewer isolates but a high prevalence of AMR and VAGs. Further studies are needed to elucidate the epidemiology of these virulence factors in the pathogenesis of *A.* *butzleri*.

In previous studies, ERIC-PCR has been used to determine the molecular etiology of arcobacters isolated from various sources such as poultry [6, 37], cattle [55] and food [36]. ERIC-PCR based fingerprinting was used to genotype *A. butzleri* strains isolated from different agricultural sites. Dendrogram analysis and clustering pattern revealed homogeneity among some of the *A. butzleri* isolates collected from sites 1, 9 and 15. However, some of the *A. butzleri* isolates from sites 15 and 23 did not cluster with other isolates, indicating the possibility of heterogeneity among these isolates. High genetic similarity was seen among the isolates from the same site. The results of this study showed a significant genetic similarity among isolates, highlighting a common source of contamination. These results are in congruency with previously reported data where low variability was observed in *A. butzleri* isolates from a dairy plant [56]. The analysis suggests that the water at these sites could be fecally contaminated [57] supported by previous studies linking the presence of *Arcobacter* species in water to high levels of fecal contamination [58, 59].

Seven isolates from site 9 and four from site 15 displayed identical ERIC-PCR patterns and 100% homogeneity in cluster analysis, with similar AMR and VAGs profiles. Similarly, five isolates from site 1 showed 100% homogeneity to three isolates from site 6, showing similar AMR and VAGs profiles. These results reveal a close genetic relationship between isolates from sites 9 and 15, and sites 1 and 6, suggesting a common origin. Although site 9 is hydrologically disconnected from site 15 and site 6 has water that could feed site 1, over long distances and under significant dilution, inputs from other sources, including runoff and wildlife (migratory birds), may contribute to the prevalence of *A. butzleri* strains in these sites. All isolates with similar ERIC-PCR patterns, cluster analysis homogeneity, and similar AMR and VAGs profiles were potentially have a shared origin and possibly more pathogenic or resistant. This study concludes that the presence of AMR and VAGs may increase the resistance and pathogenicity of strains, posing potential health risks to humans and animals.

## Conclusions

In conclusion, this study has provided insightes into the prevalence, distribution, and co-occurrence of AMR and VAGs among *A. butzleri* strains isolated from eleven stream sites within a mixed-use but predominately agricultural river basin in eastern Ontario Canada. The high frequency of MDR in isolates from different sites indicates potential public health risks. Moreover, susceptibility to ciprofloxacin, gentamycin, erythromycin, and tetracycline can be considered drugs of choice in agriculture and animal husbandry. The lack of standardized procedures to determine antibiotic susceptibility patterns underscores the urgent need for developing such testing methods. The majority of the isolates were detected with *cia*B, *mvi*N, *tly*A and *pld*A VAGs, necessitating further investigation into their pathogenic roles. A positive correlation between AMR and VAGs in *A. butzleri* isolates raises concerns about increased resistance and pathogenicity. Furthermore, given the pathogenic potential of these isolates, contaminated water at these sites may serve as a plausible transmission route of virulent strains to humans and animals.

## Methods

### Water sampling, isolation, and culturing

**Fig. 2:**
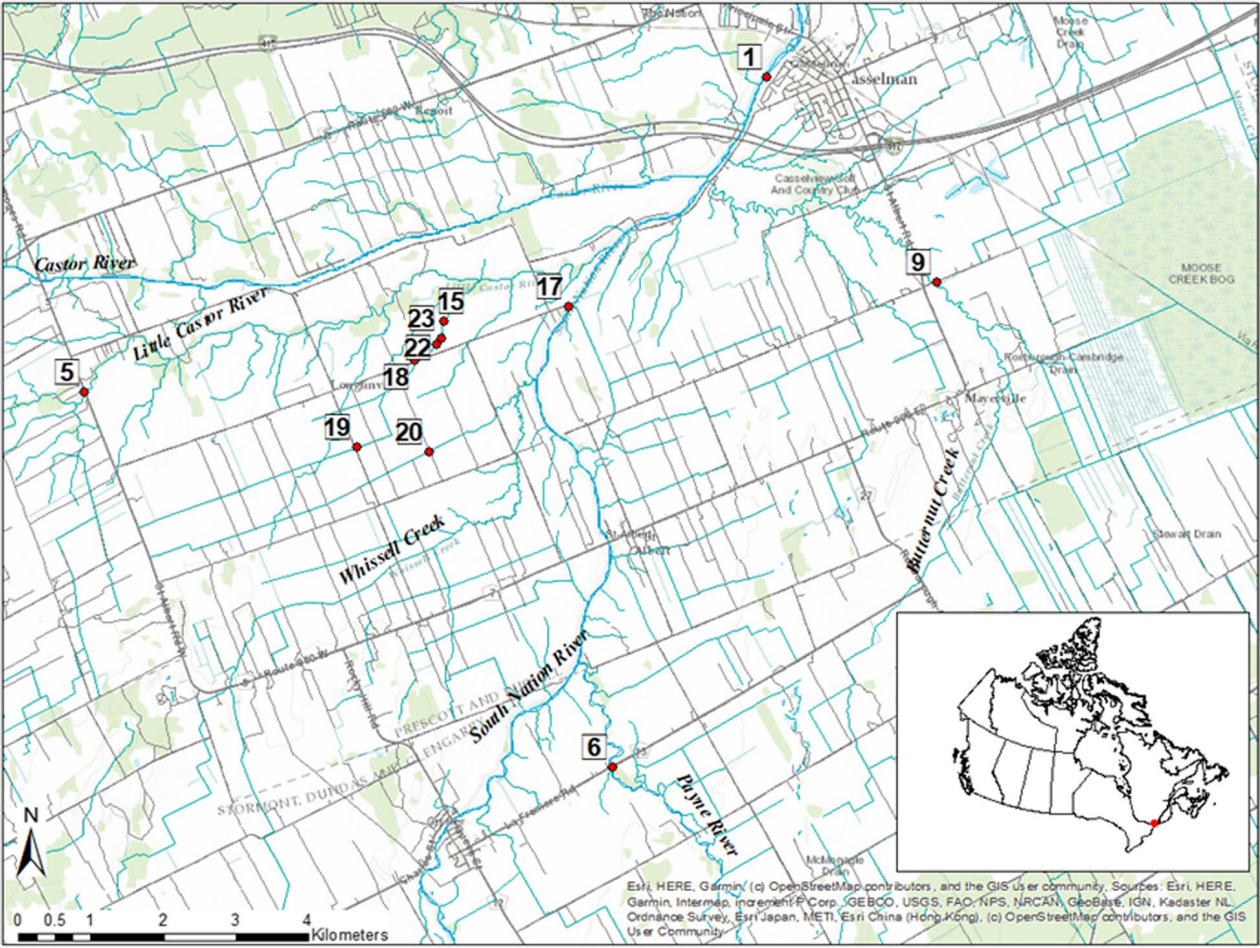
Map showing agricultural watershed surface water sampling sites selected for the study

A total number of 797 surface water samples were collected from 11 different water sampling sites (sites designated as 1, 5, 6, 9, 15, 17–20, 22 and 23) located in the South Nation River basin near Ottawa, Ontario, Canada (Fig. 2). A detail watershed description (Table 6) is previously given by Wilkes et al. [57, 60]. The sample collection was carried out on a bi-weekly basis between May to November 2012 to 2019. The samples were collected in sterile bottles, placed on ice in a cooler, transported to the AAFC-Ottawa laboratory, and processed within 24 h of their collection for microbiological analyses. For the isolation of *Arcobacter*, the samples were processed according to the previously described procedure [61]. Briefly, 1 mL of water sample was resuspended in 9 mL of peptone water (PW) using a single ten-fold serial (ranging from 10^–1^ to 10^–6^) dilution. A 100 µL of each sample was plated on *Arcobacter* selective isolation agar (ASIA) media (Oxoid, Nepean, ON) containing antibiotics supplements; fluorouracil, amphotericin-B, cefoperazone, novobiocin, and trimethoprim. The plates were incubated for three to six days at 30 °C under microaerophilic (85% N_2_, 10% CO_2_ and 5% O_2_) conditions.

**Table 6:** Land use description of each site sampled for isolation of *A. butzleri* in this study

Sampling Site	Strahler Stream Order	Agriculture (% land use)	Forest/wetland (% land use)	Urban (% land use)
1	7	51.58	44.33	2.40
5	4	65.00	31.15	1.85
6	5	54.20	43.67	1.46
9	4	71.71	26.66	0.73
15	2	90.16	9.75	0.09
17	6	49.78	48.13	1.23
18	2	89.87	10.03	0.10
19	2	88.82	11.03	0.15
20	2	90.24	9.04	0.04
22	2	89.79	10.11	0.10
23	2	89.97	9.94	0.09

Based on colony morphology, a putative *Arcobacter* single colony was sub-cultured on modified Agarised Arcobacter Medium (m-AAM) containing selective antibiotic supplements; cefoperazone, amphotericin-B, and teicoplanin. The plates were incubated according to the conditions mentioned above. The putative culture isolates were further confirmed by Gram staining reaction and PCR assays designed specifically for the *Arcobacter* genus and *A. butzleri* species, respectively.

For this study, *A. butzleri* ATCC 49616, *A. cryaerophilus* NCTC 11885, and *A. skirrowii* ATCC 51132 reference strains were used as positive and negative controls. The strains were also grown on selective growth media according to the specified culture conditions mentioned above.

### Nucleic acid extraction

The DNA extraction from *Arcobacter* culture isolates, recovered from water samples, were prepared by re-suspending a purified single colony in a sterile 1.5 mL microfuge tube containing 100 μL TE buffer (10 mM Tris–HCl and 1 mM EDTA, pH 8.0). The cells were gently mixed and boiled for 10 min, as described by Khan et al. [62]. The tube was centrifuged at high speed for 60 s., and the supernatant containing purified DNA was used for subsequent examination and quantification by agarose gel electrophoresis and an ND-1000 spectrophotometer using low-range quantitative DNA marker (Fisher Scientific, Ottawa, ON), respectively. The DNA extract was stored at -20 °C for further PCR analyses.

### *Arcobacter* genus and species-specific multiplex PCR assays

To identify *Arcobacter* isolates, a DNA-based PCR amplification assay was performed using *Arcobacter* genus-specific oligonucleotide primers and PCR protocol previously described by Harmon and Wesley [63]. The PCR reaction was performed in a 25 µL volume containing 5.0 ng of template DNA, 50 pmol each of *Arcobacter* 16S rRNA gene primer pair (Table 7), 1.25 U of Taq DNA polymerase 1 × buffer with MgCl_2_, 200 μM each of the dNTPs (Fisher Scientific, Nepean, ON). The PCR reaction was performed in a Mastercycler Gradient PCR system (Eppendorf, Hauppauge, NY) using an initial denaturation (94 °C for 4 min), followed by 25 amplification cycles consisted of denaturation: 94 °C for 60 s; annealing: 56 °C for 60 s.; extension: 72 °C for 60 s. with final extension cycle at 72 °C for 7 min.

**Table 7:** List of *Arcobacter* genus and species-species oligonucleotide PCR primer sequences used in this study

*Arcobacter* spp.	Target gene	Sequences (5´-3´)	size (bp)	Reference
Genus	16S rRNA	AGA GAT TAG CCT GTA TTG TAT C TAG CAT CCC CGC TTC GAA TGA	1223	Harmon and Wesley [63]
*A. butzleri*	16S rRNA	GCA CAT TCT ATT TTC AAA GAA GGG GAA TGG GTT ATT AAA CTC TGC	654	Khan et al. [62]
*A. lanthieri*	*gyr*B	CAG CTT TAT GTG AAG TTG TAG C TGC CTT TTT AGC AGC TTC TC	461	
*A. faecis*	*cpn*60	GCT CCA GGA AGT ACA AAA GTA G AGG CTA GCA GCT ACT CCC	372	
*A. skirrowii*	*gyr*A	GGC GAT TTA CTG GAA CAC A CGT ATT CAC CGT AGC ATA GC	262	
*A. cryaerophilus*	*rpo*B	AGT TCT GAA GCA ATA GAT TTA ATG G CTG CAA TTC CTT CGA TTT GC	152	
*A. cibarius*	*gyr*A	GCA CAA TCT AGG GGA ACT T CAA ATC AAG GGC TTC AGC AC	72	

Furthermore, *A. butzleri*, *A. cryaerophilus*, *A. cibarius*, *A. faecis*, *A. lanthieri*, and *A. skirrowii* were identified using species-specific multiplex PCR protocol described by Khan et al. [62]. The PCR amplification reaction was carried out in a 25 μL reaction mixture containing 1 × buffer with MgCl_2_, 200 μmol L^−1^ each of the dNTPs (Fisher Scientific, Nepean, ON, Canada), 1 U of Ex-Taq DNA polymerase, 0.1 μmol L^−1^ of *A. butzleri*, 0.2 μmol L^−1^ of *A. lanthieri* and *A. faecis*, 0.3 μmol L^−1^ of *A. skirrowii* and *A. cryaerophilus* and 0.4 μmol L^−1^ of *A. cibarius* primer pairs (Table 7) and 10 ng of each target DNA template. The PCR protocol was performed with an initial denaturation (94 °C for 3 min) followed by 35 cycles consisted of denaturation (94 °C for 30 s), annealing (58 °C for 30 s), and extension (72 °C for 30 s) with a final extension at 72 °C for 5 min.

The amplified products were electrophoresed on a 1.5% agarose gel and stained with ethidium bromide (0.5 µg mL^−1^), visualized under an ultraviolet (UV) transilluminator, and photographed using an Alpha Imager (Fisher Scientific) gel documentation system.

### Antimicrobial resistance analysis

Antimicrobial susceptibility to eight antibiotics (three concentrations of each), including azithromycin (Azi; 4, 8, 16 μg mL^−1^;), chloramphenicol (Chl; 16, 32, 64 μg mL^−1^), ciprofloxacin (Cip; 2, 4, 8 μg mL^−1^), clindamycin (Cli; 4, 8, 16 μg mL^−1^), erythromycin (Ery; 16, 32, 64 μg mL^−1^), gentamycin (Gen; 4, 8, 16 μg mL^−1^), nalidixic acid (NaI; 32, 64, 128 μg mL^−1^), and tetracycline (Tet; 8, 16, 32 μg mL^−1^) obtained from Sigma-Aldrich (ON, Canada) was determined based on the criteria described by CIPARS and standards of the Clinical and Laboratory Standards Institute [64] at breakpoint concentration commonly used for *Campylobacter* species since no breakpoint values for *Arcobacter* spp. are available [65]. The agar dilution method, as described by Gaudreau and Gilbert [66], was applied. Briefly, *A. butzleri* strains were incubated overnight in a 96-well microplate with 200 μL per well of *Arcobacter* medium (AM) broth at 30 °C under microaerophilic conditions. The cells were then transferred to the surface of rectangular Mueller–Hinton (MH) agar plates by a 96-floating pin replicator (V&P Scientific, San Diego, CA). Agar plates were incubated at 30 ºC under microaerophilic conditions for 3 days, and the growth of *A. butzleri* isolates on plates with antimicrobials was compared to their growth on control plates without antimicrobial agents. The results were interpreted in accordance with the CLSI [64].

### Multiplex PCR assays for detection of virulence-associated genes (VAGs)

*A. butzleri* culture isolates were analyzed for the presence of VAGs using our previously developed three multiplex PCR-based assays. Each of the assays was designed to detect: 1). *cia*B and *cj*1349, 2). *pld*A, *irg*A and *hec*A, or 3). *tly*A, *mvi*N and *hec*B genes, respectively [67]. Each multiplex PCR (mPCR) amplification assay was carried out in 25 μL of reaction mixtures containing 1 μL (50–70 ng μL^−1^) of template DNA, 1 U of Ex Taq DNA polymerase (Fisher Scientific), and the compatible PCR reagents, including 1 × buffer with MgCl_2_, 200 µM each of the dNTPs, and 0.1 µM of each set of forward and reverse primer pair (Table 8). A total volume of 25 µL was adjusted by adding nuclease-free water to the reaction mixture. All three PCR assays were carried out in a Mastercycler Gradient PCR system (Eppendorf, Hauppauge, NY) under the following standardized cycling conditions: initial denaturation at 95 °C for 4 min, 30 cycles of denaturation at 95 °C for 30 s, annealing at 56 °C for 45 s, extension at 72 °C for 45 s, and final elongation at 72 °C for 5 min. Amplified PCR products were analyzed by gel electrophoresis in 2% agarose gel using 100 bp DNA size marker (Life Technologies, Grand Island, NY). The gels were stained, scanned and photographed using the procedure mentioned in the preceding section.

**Table 8:** List of virulence-associated genes, oligonucleotide PCR primer sequences and amplicon sizes for each multiplex PCR assay used in this study [67]

Target gene	Sequences (5´-3´)	size (bp)
*cia*B	TGG GCA GAT GTG GAT AGA GCT TGG ATA GTG CTG GTC GTC CCA CAT AAA G	284
*cj*1349	CCA GAA ATC ACT GGC TTT TGA G GGG CAT AAG TTA GAT GAG GTT CC	659
*pld*A	TTG ACG AGA CAA TAA GTG CAG CCG T CTT TAT CTT TGC TTT CAG GGA	293
*irg*A	TGC AGA GGA TAC TTG GAG CGT AAC T GTA TAA CCC CAT TGA TGA GGA GCA	437
*hec*A	GTG GAA GTA CAA CGA TAG CAG GCT C GTC TGT TTT AGT TGC TCT GCA CTC	537
*tly*A	CAA AGT CGA AAC AAA GCG ACT G TCC ACC AGT GCT ACT TCC TAT A	230
*mvi*N	TGC ACT TGT TGC AAA ACG GTG TGC TGA TGG AGC TTT TAC GCA AGC	294
*hec*B	CTA AAC TCT ACA AAT CGT GC CTT TTG AGT GTT GAC CTC	528

## Enterobacterial repetitive intergenic consensus-polymerase chain reaction (ERIC-PCR) assay

To assess the genetic diversity of *A. butzleri* isolates, ERIC-PCR assay was carried out using method described by Houf et al. [68]. Briefly, the PCR was performed in a mixture containing a total volume of 25 mL containing 5 mL 5 × PCR buffer, 4 mM MgCl_2_, 10 pmol uL^−1^ primers ERIC-F2 (5´-ATG TAA GCT CCT GGG GAT TCA C-3´) and ERIC-R1 (5´-AAG TAA GTG ACT GGG GTG AGC G-3´), 5U Taq polymerase and 10 uL (2 ng) of DNA template. DNA amplification consisted of an initial denaturation at 94 °C for 2.5 min, followed by 40 cycles of amplification (denaturation at 94 ºC for 1 min, annealing at 25 ºC for 1 min and extension at 72 ºC for 2 min) ending with final elongation at 72 ºC for 7 min. The amplified products were analyzed by electrophoresis in a 2% (w/v) 1xTAE agarose gel at 100 V for 2.5 h. The gels were stained, scanned and photographed using the procedure mentioned in the preceding section.

The ERIC-PCR patterns were analyzed by constructing dendrograms using the Gel Compar-II program (Applied Maths BVBA, Kortrijk, Belgium) where all isolates obtained in this study were grouped into different clusters and clades.

### Statistical analysis

Statistical analyses, including McNemar Chi-square Contingency and Fisher's Exact Tests, were applied to compare the rate of prevalence, antimicrobial resistance (AMR), virulence-associated genes (VAGs) and genotypic properties in *A. butzleri* strains across various sampling sites and to determine the pairwise association and co-occurrence of the AMR, VAGs, and genetic determinants in *A. butzleri* strains using STATISTICA 10.0 [69]. A *p*-value of < 0.05 was considered statistically significant. The discriminatory index of ERIC-PCR was calculated based on Simpson's Index of Diversity as described by Hunter and Gaston [70].

## Data Availability

The material and dataset used and analyzed in this study are added to the manuscript. There is no additional data available from the authors.

## Abbreviations

AMR: Antimicrobial Resistance
VAGs: Virulence-associated genes
ERIC-PCR: Enterobacterial Repetitive Intergenic Consensus-Polymerase Chain Reaction
MDR: Multidrug Resistance
PW: Peptone Water
m-AAM: Modified Agarised Arcobacter Medium
